# Development of a Tetherless Bioimpedance Device That Uses Morphologic Changes to Predict Blood Flow Restrictions Mimicking Peripheral Artery Disease Progression

**DOI:** 10.3390/bios14060286

**Published:** 2024-06-01

**Authors:** Sungcheol Hong, Gerard Coté

**Affiliations:** 1 Department of Biomedical Engineering, Texas A&M University, College Station, TX 77843, USA; gcote@tamu.edu; 2 Department of Electrical Engineering, Texas A&M University, College Station, TX 77843, USA; 3 Center for Remote Health Technologies and Systems, Texas A&M Engineering Experiment Station, Texas A&M University, College Station, TX 77843, USA

**Keywords:** arterial pulse monitoring, bioimpedance device, diagnostic variability, peripheral artery disease (PAD), point-of-care, wearable vital sign monitoring

## Abstract

A tetherless multi-targeted bioimpedance device was designed, modeled, built, and tested for measuring arterial pulse and, using morphological analysis, its potential for monitoring blood flow restrictions that mimic Peripheral Artery Disease (PAD) was assessed across multiple peripheral arteries. Specifically, we first developed a small form factor, tetherless, bioimpedance device, based on high-frequency structure simulator (HFSS) simulations. After designing and building the device we then tested it in vivo on human subjects on multiple arteries and found that we did not need to modify the gain on the device compared to the bench top system. Further, it was found that changes in the morphology of the bioimpedance signal over time, depicted through the ratio of the first and second harmonic in the signal frequency, could be used to predict blood flow restrictions that mimic peripheral artery disease (PAD). The HFSS simulations helped guide the modulation frequency selection and the placement of the bioimpedance electrodes. We built the device and compared it to two commercially available bioimpedance devices and it was shown to demonstrate a distinct advantage in its multi-target capability, enabling more accurate pulse measurements from different arteries without the need for tuning the circuit for each artery. Comparing the ratio of the 1st and 2nd harmonics as a function of the blood flow restriction, the two commercial devices showed a maximum error across arteries of between 22% and 27% depending on the measurement location, whereas our system consistently displayed a stable value of just below 4%. With this system, there is the potential for comprehensive and personalized medical examinations for PAD at the point of care (POC).

## 1. Introduction

Peripheral artery disease (PAD) is a prevalent and serious vascular condition characterized by narrowed or blocked arteries that supply blood to the extremities, primarily the legs or arms [1,2]. Globally, in 2019, over 113 million people lived with PAD and over 10 million new cases occurred, resulting in 74 thousand deaths, 500 thousand years lived with disability, and over 1 million years of life lost [3].

Bioimpedance has been traditionally used to measure body composition (e.g., water, fat, muscle) but has also been used for a number of cardiovascular applications including multichannel bioimpedance for detecting vascular tone or compliance in human limbs [4,5], leg bioimpedance as a prediction of heart failure [6], monitoring blood pressure with pulse wave velocity [7], modeling the effects of vascular disease on bioimpedance [8], and general use of bioimpedance for cardiovascular disease (CVD) [9]. However, we could not find any articles that describe using bioimpedance for peripheral artery disease. Rather, the tests currently used to diagnose PAD can include angiography using X-rays, MRI or CT [10] or blood tests [11]. In addition, often bedside tests are conducted including the ankle-brachial index (ABI) test, toe-branchial index (TBI), toe pressure (TP), or continuous wave Doppler [12].

Each of these approaches has inherent limitations. MRI and CT scans, while providing objective measurements, may be limited by factors such as cost, accessibility [13,14,15,16], and potential exposure to ionizing radiation [17,18,19]. On the other hand, ABI, TBI, and TP rely on the subjective judgment of healthcare professionals, introducing a potential limitation in terms of the quantitative assessment of the conditions [20,21,22,23,24]. Thus, in this paper, we describe a tetherless multi-targeted bioimpedance point-of-care bedside and potentially home monitoring system to monitor PAD through morphological analysis of the pulse, which includes robust data acquisition of signals regardless of the measurement site. Table 1 includes a comparative table for state-of-the-art techniques in similar domains.

Existing bioimpedance pulse devices allow measurements from various locations, but the signal processing (either analog or digital) typically needs to be customized for each specific vessel investigated due to the different morphological features of the pulse [25,26,27,28,29,30]. The result of this is that, even though it is not difficult to extract signals from various arteries using existing commercial devices, it is challenging to quantitatively compare waveforms from different areas due to the need to adjust settings for each specific artery [31]. Although a cleaner measurement is potentially possible by adjusting the settings of both the commercial systems and our system for each artery, the focus of this study is comparing the ratio of the first and second harmonics of the signals to predict the arterial condition. In such a scenario, quantitatively comparing signals obtained from different setups would lead to reduced reliability and the numerical values themselves may vary significantly [32]. Thus, for each system, we applied common settings within that system that were optimized for the radial artery. Specifically, for our system the signal conditioning circuits used the same signal settings for the measurements at three different locations (Radial artery, Brachial artery, and Anterior Tibial artery), allowing uniform and comparable morphological features at each measurement site. Additionally, as detailed in the Section 3 below, to find the optimized injection frequency for monitoring PAD at each site, we conducted High-Frequency Structure Simulator (HFSS) simulations. Previous experiments including the Cole model, showed higher frequencies enable better tissue penetration indicating that higher injection frequencies are favorable in terms of impedance [33,34]. One group found the frequency where the pulse amplitude is high at 50 kHz was suitable for bioimpedance pulse measurements [35]. In order to expand upon these results, we ran an HFSS simulation to find the most suitable injection frequency that achieves high vascular selectivity based on the electrical characteristics of tissues including the epidermis, dermis, artery, vessel, and electrodes [36]. Specifically, we simulated injecting current at various frequencies to determine which frequency provided the highest vascular selectivity. We also assessed at which frequency the current concentration was highest in the blood vessels and which did not penetrate too deeply beneath the vessels. We also confirmed how the signal amplitude progressed by conducting HFSS simulations to predict the signal intensity of the sensor as the plaque size in the blood vessels increased. Furthermore, we determined the sensitivity to plaque growth at various injection frequencies to confirm whether the selected frequency was optimized for PAD monitoring. Additionally, we checked whether the injected current flow remained relatively stable even when the four electrodes were slightly misaligned, ensuring practical applicability under real-world conditions. 

After determining the frequency conditions and building the system and common signal processing algorithms we tested the system in vivo. Specifically, we measured the flow at three locations and used a blood flow restriction (BFR) band proximal to the radial artery to mimic the reduced flow seen in PAD [37,38]. The results were compared with Doppler velocimetry studies conducted in the context of PAD’s pathophysiological approach [39], especially in terms of the features of signal shape. For cross-validation of the PAD-mimicking experiment, we compared the predicted signals as the flow was restricted with the BFR band, mimicking PAD progression. Subsequently, we demonstrated how to quantitatively assess these morphological features and presented their potential applicability for PAD monitoring. We conducted a quantitative comparison of the results based on this approach. Additionally, we demonstrated the possibility of performing a quantitative analysis of morphological features in all three arteries (Radial artery, Brachial artery, and Anterior Tibial artery). In light of the challenges and limitations in existing PAD monitoring methods, our research suggests a solution that combines bioimpedance technology and morphological analysis for comprehensive and convenient PAD assessment. Our proposed tetherless multi-targeted bioimpedance device offers a novel approach to monitoring arterial conditions, potentially allowing patients and healthcare providers to acquire pulse data at specific arterial points without the constraints of location or signal customization. This contribution not only provides a new potential dimension to PAD monitoring but also opens the door to predictive and personalized healthcare solutions at the point of care. Specifically, one could envision that like an ankle-brachial index test in a clinic rather than taking the blood pressure using a cuff on the upper arm (Brachial artery) and ankle and then calculating the ratio, a person could wear the bioimpedance device on the arm or ankle or both and obtain real-time harmonic ratio measurements that would be sent via Bluetooth to a personal device for analysis via an app to calculate whether you have PAD or early PAD onset, or to track PAD severity over time, providing the health care provider with information needed for clinical decision making.

## 2. Materials and Methods

### 2.1. System Illustration and Target Locations on the Body

**Figure 1:**
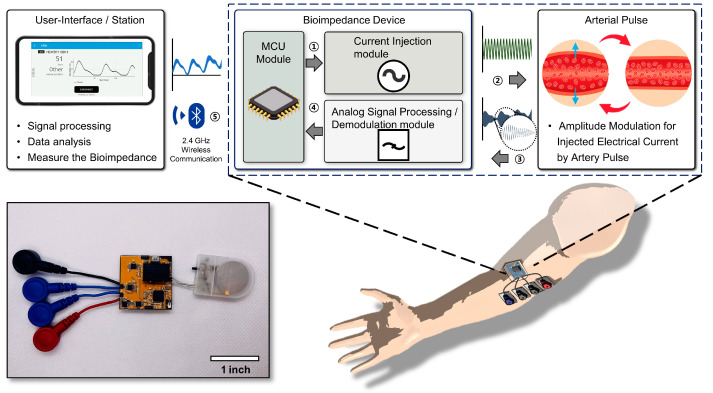
General overview illustration for the entire wireless multi-target bioimpedance system.

As depicted in Figure 1, we designed and built a flexible and tetherless wearable bioimpedance system powered by coin batteries with a voltage of 3.3 V on a small flexible printed circuit board (fPCB) circuit board of approximately 1.2 inches by 1.2 inches (excluding the battery). The system can transmit signals through Bluetooth Low Energy (BLE). Additionally, the signal adjustment function was developed to help to more stably measure arterial heartbeats in various locations.

An overview of the system and the actuation mechanism of the multi-targeted PAD monitoring wearable bioimpedance system is depicted in Figure 1. The system operates with a Bluetooth Low Energy Microcontroller Unit (MCU) [40]. Initially, the MCU (NRF52832, Nordic Semiconductor, Trondheim, Norway) generates a sinusoidal waveform using pulse width modulation (PWM), which is then rectified and supplied as a sinusoidal current to the body through the revised Howland current pump [41]. When amplitude modulation (AM) occurs due to changes in impedance caused by the pulse of the artery [42,43] the signal is then received by the demodulation module of the device through the electrodes. A multiplication process with a carrier frequency similar to traditional AM demodulation is performed [44], followed by filtering and signal adjustment before entering the MCU’s internal analog-to-digital converter (ADC) through the analog input pin. The reconstructed pulse signal is then transmitted to the user’s smartphone through the antenna, enabling real-time monitoring, signal processing, and data interpretation on the phone.

**Figure 2:**
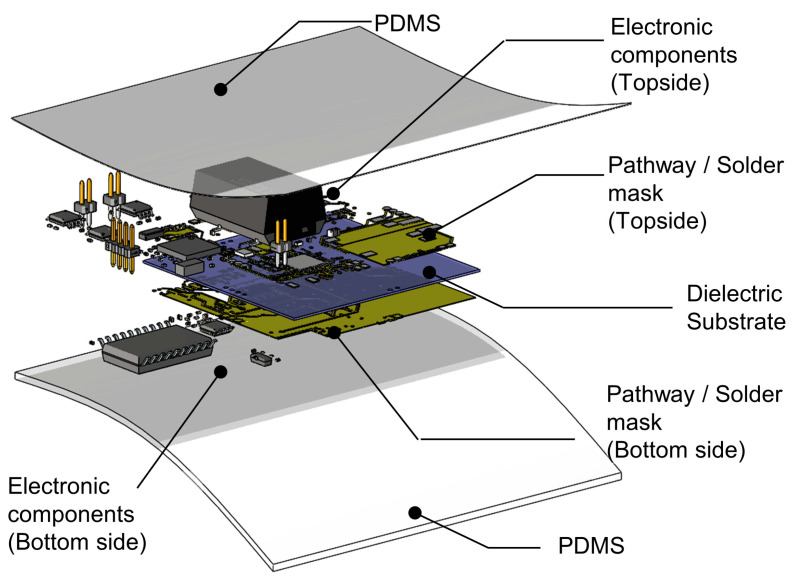
Illustration of the internal structure of the device in an exploded view.

The exploded view of the device used in this study is shown in Figure 2. The top and bottom layers are coated with Polydimethylsiloxane (PDMS) to mitigate heat dissipation and prevent corrosion due to sweat [45], and the electrical components are arranged on both sides of the double-sided fPCB. PDMS (Dow^®^ (Midland, MI, USA), SylgardTM 184 Silicone Elastomer Kit) has been used with a 1:10 ratio [46].

**Figure 3:**
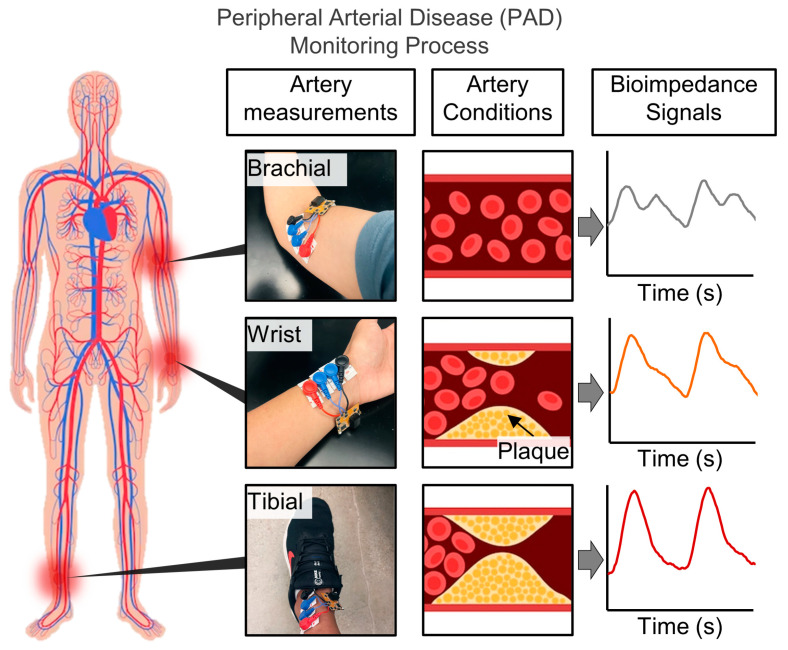
Examples of the versatile locations in which the device can be placed and representative methods of monitoring peripheral arterial disease.

Figure 3 shows an example of various arteries that the system can measure. In this study, measurements were taken from the radial artery, brachial artery, and anterior tibial artery to emphasize the ability to monitor vascular conditions in various locations without the need for separate tuning or signal processing. The selection of the radial artery was based on its widespread recognition as a common and non-invasive method for measuring arterial properties in peripheral blood vessels [47]. Additionally, the Brachial artery and Tibial artery were chosen due to their involvement in the Ankle Brachial Index (ABI Test), a diagnostic tool used to assess Peripheral Artery Disease (PAD) by measuring systolic pressure [48]. Commercial software Altium Version 20.0.10 (Build 225, Altium Ltd. (San Diego, CA, USA)), has been used for designing the electrical schematics and PCB artwork.

### 2.2. Bioimpedance Circuit and Node-by-Node Signal Examples

**Figure 4:**
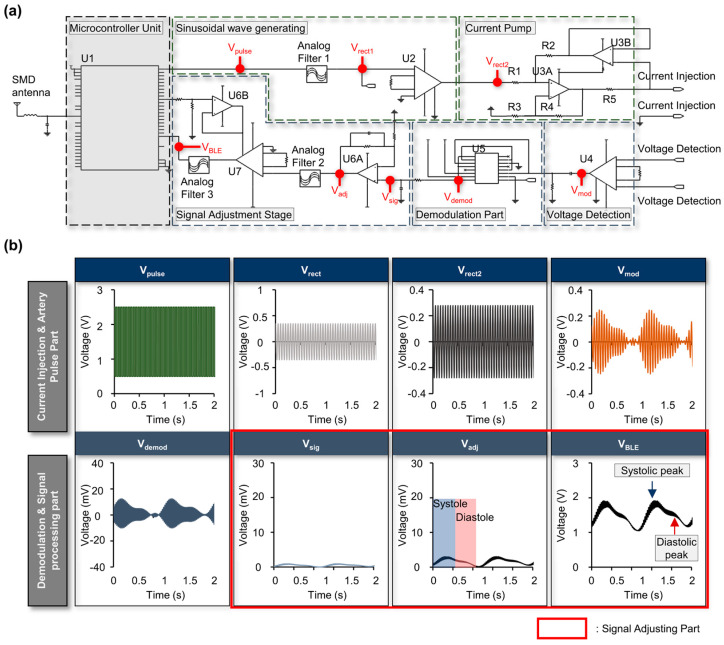
Electrical circuit characteristics of the multi–target bioimpedance circuit. (**a**) Circuit schematic of multi-target bioimpedance circuit (U1 = NRF52832 (MCU), U5 = AD630 (Multiplier), U2, U4, U7 = INA823, U3, U7 = OPA2387, R1, R2, R3, R4 = 10 kΩ, R5 = 2 kΩ). (**b**) Representative signal flow for the circuit from each point.

Figure 4 shows the overall characteristics of each node from a circuit perspective. While there are commercially available ICs supporting bioimpedance in the market such as the MAX3000x, or AD594x (Analog Devices, Inc. (Wilmington, MA, USA)) [49,50,51,52,53,54,55], our research took a direct design approach to address issues related to phase differences of local oscillators that may arise and be problematic when simultaneously measuring various points for the future research [56,57,58]. Figure 4a illustrates the circuit and important nodes, while Figure 4b depicts representative images of how the signal changes at those crucial nodes. We also designed the circuit from scratch to allow us to tailor the signal to fit into the acquisition window of the MCU. When a signal, Vpulse, a sinusoidal wave with the same frequency as the injection frequency, is generated through the MCU, it is converted to a sine wave with an offset of 0 through Analog Filter 1 (Figure 4a). Using a Wien Bridge oscillator, a cleaner sinusoidal wave can be produced compared to using the MCU’s PWM [59], but it is difficult to create an accurate frequency with this approach and the frequency characteristics can change depending on the op-amp. Moreover, the passive components’ error rate can cause the operating frequency to change or not work at all [60] (Supplementary Figure S1). Therefore, in this design, a filtering method using PWM was used instead. The purpose of analog filter 1 shown in Figure 4a is to convert the PWM signal into a sinusoidal waveform. By doing so, it is possible to eliminate the second and third harmonic components inherent in the PWM signal and utilize only the first harmonic for amplitude modulation. The objective was to maximize signal integrity by incorporating the minimum number of frequency components in AM, which utilizes nonlinearity. 

The filter allows only the 10 kHz sinusoidal signal to pass through to the next stage by filtering out any other frequencies. This signal is then amplified by the Instrumentation Amplifier (INA) and enters the revised Howland current pump, which supplies a constant amount of current effectively independent of changes in the impedance of the targeted load [61]. The Howland current pump used in this study includes a total of five resistors, with the resistor located at the output designated as R5 and the others including R1, R2, R3, and R4 are all set equal to each other. Using this arrangement, the output supplies a constant current to the target equal to one-fifth of the input voltage regardless of the load impedance. In this case, changes in impedance due to variations in biological tissue will not affect the amount of injected current by the current pump. Although the original Howland Current Pump used one op-amp [41,61,62,63], it is more dependent on the impedance change in the subject and has a relatively low output impedance, Z_load_. Therefore, the modified method was used in this paper. By employing the improved current pump, the injected current remains more independent from load variations. Additionally, through the extension of the output impedance across all frequency domains, the dependency on both the source and load has been minimized (Supplementary Figure S2).

The signal returning through the Artery is fed into the INA stage and becomes V_mod_, which is split into the Carrier frequency component, twice the Carrier frequency, and the desired signal component through a multiplier. The signal is then adjusted using an analog RC filter. Analog Filter 2, which is a bandpass filter, filters out noise except for signals in the range of 0.3 Hz to 3 Hz [64]. Analog Filter 3 is a low-pass filter with a cutoff frequency of 3 Hz. As a last stage, the offset and amplitude of the signal are adjusted so that the signal can fit into the MCU internal ADC acquisition window, as V_BLE_, which is the input signal to the MCU (Figure 4a,b). Detailed information about the circuit including the Bill of Materials (BOM) is in Supplementary Figures S3–S10. Commercial software OrCAD Capture (PSpice Plugin v16-5-13B, Cadence (San Jose, CA, USA)) was used for the electrical circuit simulation. The simulation was run with a relative accuracy of the voltages and currents of 0.001. The best accuracy of voltages, currents, and charges are 0.1 µV, 1.0 pA, and 0.01 pC, respectively. The minimum conductance for any branch was 1.0^−12^/ohm. The DC bias ’blind’ iteration limit was 150, and the DC bias ’best guess’ iteration limit was 20. The transient time point iteration limit was set as 10. For all the simulations, the normal default temperature was 27.0 °C. In addition, the auto converges function was used.

**Figure 5:**
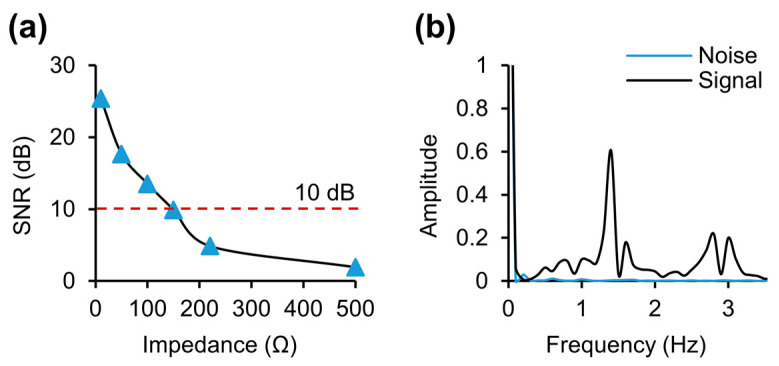
SNR evaluation of circuits and systems. (**a**) Evaluation of SNR by comparing the standard deviation of DC and signals when static resistance is connected. (**b**) SNR evaluation when the load is open and when measuring signals from the artery.

The results of the signal–noise ratio (SNR) evaluation of the circuit are depicted in Figure 5. In the case of Figure 5a, a static resistor was connected to the load for the evaluation of static resistance, and the offset value was compared with the noise.

SNR evaluation was also conducted by comparing the peak values when the load site was open and when measuring the actual arterial signal. The resulting SNR was 34.84 dB. Generally, maintaining an SNR above 10 dB at impedance values below 100 ohms is considered acceptable for the reliability of DC noise in consideration of typical bioimpedance values and, thus, it can be observed that the SNR is very good (Figure 5b).

## 3. Results

### 3.1. HFSS Simulation for Optimization

**Figure 6:**
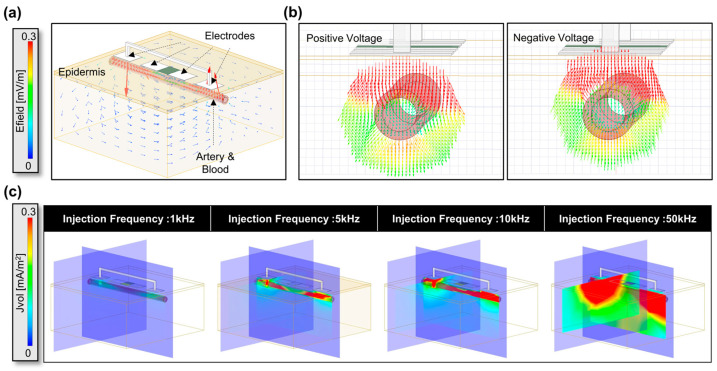
Electrical HFSS simulation results for the Multi-target Bioimpedance Device showing; (**a**) HFSS simulation model with electrodes and tissue layers (epidermis, dermis, artery, and blood) including the E field distribution shown upon current injection. (**b**) Cross-sectional view of the E field propagation into the artery upon current injection. (**c**) Comparison of current flow at different injection frequencies (1 kHz, 5 kHz, 10 kHz, and 50 kHz).

The results presented in Figure 6 demonstrate the outcomes of the HFSS simulations conducted to guide the modulation frequency selection and the placement of the bioimpedance electrodes to maximize the amount of signal entering and coming from the artery rather than the non-arterial tissue as well as to assess the effect of misalignment of the electrodes relative to the artery. In Figure 6a, the simulation setup includes the model with four electrodes: two current injection probes and two voltage detection probes [65,66,67]. The model also incorporates layers representing the epidermis, dermis, artery, and blood. The electrical field (E field) vector in the model can be observed when current is injected into the skin. Figure 6b provides a cross-sectional view showing the flow of the electrical field toward the artery after the current injection. We can observe the sinusoidal differential signal swing while passing through the skin and that the field drifts accordingly. Moreover, as the injected current traverses through the blood vessels, the field intensity weakens beneath the artery, indicating that most of the current is along the blood vessels rather than simply passing beneath them and going deeply into the skin. This is further illustrated by the images in Figure 6c, which clearly shows the current within the blood vessels. Specifically, to investigate the impact of different injection frequencies, simulations were performed at 1 kHz, 5 kHz, 10 kHz, and 50 kHz, as shown in Figure 6c. As the frequency increases, the current strength also increases due to the lower impedance at higher frequencies [34,68]. Notably, at 1 kHz and 5 kHz, the current flows more evenly, while at 50 kHz, although the current flows stronger than at 10 kHz, it penetrates deeper beneath the artery and thus probes more of the tissue rather than the artery. This finding suggests that the optimal frequency range for the device needs to consider the impedance changes primarily coming from the artery while minimizing the signal entering from the non-arterial tissue. We also examined the influence of the model’s feature size and location on the signal. As shown in Supplementary Figure S11, the depth or position of the artery has relatively little impact on the signal, whereas thinner skin thickness, specifically as you go below 1 mm thickness allows the signal to penetrate more effectively. 

**Figure 7:**
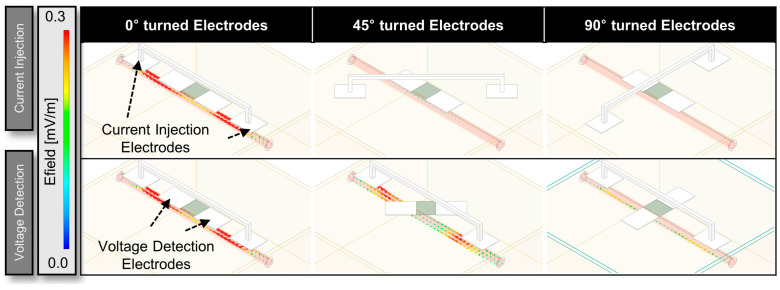
HFSS simulations for different electrode array angles (0°, 45°, and 90°) to assess the output as a function of the configuration.

Figure 7 focuses on the analysis of the electrode array as a function of the angles of both the current injection and voltage detection electrodes relative to the artery. Specifically, the angles tested were from 0° to 90° relative to the artery for both sets of electrodes. In the experiment, we observed the highest strength of the signal when both electrode pairs were perfectly aligned at 0 degrees, which was expected. However, the key focus of our study was to determine how much misalignment is acceptable within the frequency range we set. We defined a threshold based on the point where the roll-off occurs, or in other words, where the signal drops to half its strength compared to perfect alignment. According to the simulation results, even with the maximum misalignment of 20 degrees for the current injection electrodes and 40 degrees for the voltage measurement electrodes, the field intensity still was at 52% of the field intensity compared to the case where the electrodes are perfectly aligned with the artery. Furthermore, when the injection electrodes were tilted up to 10 degrees, the signal remained at more than half of its original amplitude even when the voltage measurement part was misaligned by 90 degrees. However, when the current injection electrode was misaligned below 50 degrees, the field intensity dropped by less than 20%. The electrical characteristics of the biological tissue used in the simulation are presented in Supplementary Figure S12.

**Figure 8:**
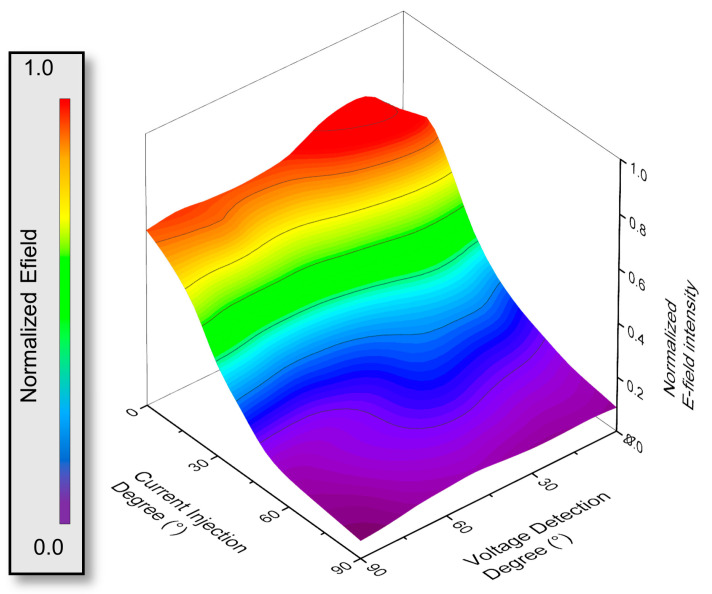
Comparison of normalized electric field intensity according to electrode misalignment.

These findings indicate that 10 kHz injection frequency can tolerate an acceptable range of misalignment, providing robust and reliable measurements in practical scenarios. By defining the acceptable threshold for misalignment, we gained insights into the device’s flexibility toward alignment for use in real-world applications, allowing for accurate artery pulse monitoring even with variations in electrode alignment (Figure 8). Further simulations were performed at various angles to explore additional possibilities (Supplementary Figure S13). Commercial software Ansys HFSS (Ansys Electromagnetics Suite 2020 R2-HFSS, Ansys (Canonsburg, PA, USA)) was used to simulate parameters and the distribution of the magnetic and electrical fields from the circuits and tissue. The conductive material was copper with a finite conductivity of 58 MS m^−1^. The substrate material for the device was set as polyamide with a relative permittivity of 4.3 and dielectric loss tangent of 0.005 [69]. The radiation region was set to 100% for +X padding, −X padding, +Y padding, −Y padding, and 300% for +Z padding and −Z padding. The power for the excitation port was set as 1 watt for better visualization in this simulation. Electrical parameters for arterial wall, skin, fat, and blood were characterized as a function of frequency [36]. The exact values are listed in Supplementary Figure S11. All dimensions for the models were taken from the M. Al-Harosh, et al. reference [70]. We did not account for changes in bioimpedance signals when blood flow was actually suppressed through the BFR band, nor did we model frequency with plaque size since these parameters were not something we controlled in our human subject study but would need to be part of a larger clinical trial.

### 3.2. System Illustration and Target Locations on the Body

**Figure 9:**
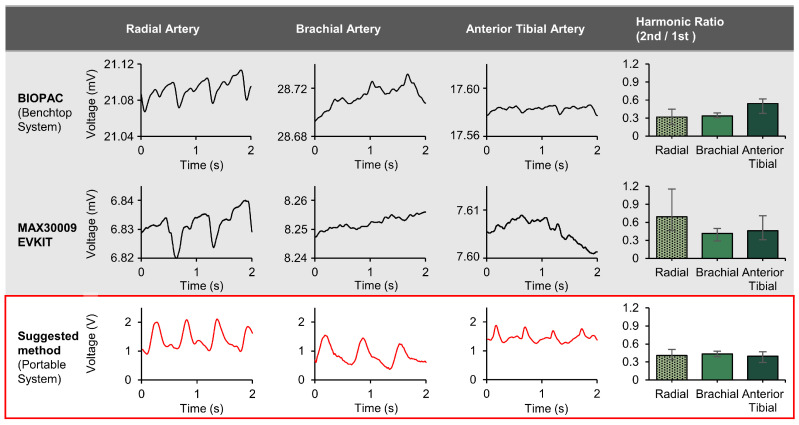
Comparison of the multi-targeted bioimpedance device with existing devices (BIOPAC, MAX30001 EVSYS) for accurate pulse measurement at multiple arterial sites (Radial, Brachial, and Anterior Tibial).

Figure 9 compares our tetherless multi-targeted bioimpedance device and two commercially available devices (BIOPAC (Goleta, CA, USA) and MAXIM MAX30001 EVSYS (Maxim Integrated, San Jose, CA)) for monitoring arterial pulse across multiple peripheral arteries that, when using morphological analysis, could monitor blood flow restrictions that mimic Peripheral Artery Disease (PAD). In all trials, the probes used were ECG Snap Electrode 3M™ Red Dot™ Monitoring Radiolucent (Model number: 408100) (Saint Paul, MN, USA). Each electrode was trimmed to a width of 1.2 cm before use. Human participant measurements were performed under the approval of the Institutional Review Board of Texas A&M University (IRB number: IRB2022-0227). 

We found that changes in the morphology of the bioimpedance signal over time, depicted through the ratio of the first and second harmonic in the signal frequency, could be used to predict blood flow restrictions that mimic PAD. We used multiple arteries since monitoring PAD typically requires observing the condition of each peripheral artery, for example, the approach is similar to comparing the ratio of ankle to brachial pressures in ABI. In this study, the system was built to provide robust phase and gain so that it could be used on multiple arteries without modifying the signal processing. It is essential to apply the same signal processing across various peripheral arteries to enable a quantitative comparison of vascular conditions. 

The device demonstrates a distinct advantage in its multi-target capability, enabling more accurate pulse measurements for different arteries without the need for tuning the circuit for each artery, which is important in order to maintain a signal shape that is not affected by a tailored signal processing circuit for each artery location and in order to perform a direct morphological comparative analysis. As noted in the introduction, the brachial artery is often greatly affected by respiration due to motion artifacts because it is close to the lungs [71,72]. Relatively, the legs were not affected much by breathing, but since the blood vessels are deeper, the absolute impedance value is higher, and the change in relative impedance due to the pulse is small [73]. To overcome such issues, the injection current can be increased, and a stronger filter applied. However, this would need to be optimized and uniformly applied since the idea is to detect artery pulses from multiple points without additional tuning, because if separate signal filters were applied to each measurement site, it would alter the shape of the signal and hinder accurate morphological comparisons between signals [74,75]. As depicted in the fourth column of Figure 9, the 1st and 2nd harmonic ratio graphs are given for each device. The experiments were conducted with five repetitions for each method and at each location on a single normal individual with no known PAD disease. After recording the signals, frequency analysis was performed to compare the peak points of the 1st and 2nd harmonic peaks in the frequency domain. This allowed us to determine the ratios between these peaks and assess the results. It can be observed that for the radial artery, brachial artery, and anterior tibial artery, the BIOPAC case shows second to first harmonic ratios of 0.318, 0.335, and 0.541, respectively for each artery. In the case of the MAXIM, the ratios are 0.693, 0.417, and 0.461. The variation in ratios, along with the difficulty in observing waveforms in areas other than the radial artery, raises doubts about the reliability of the ratios. However, when using our designed device, both the waveform shapes and the ratios (0.406, 0.435, 0.396) remain very consistent. The existing commercial devices exhibited varying harmonic ratio differences of 22% to 27% depending on the measurement site, whereas the proposed method consistently presented a stable value of just below 4% across measurement sites. This highlights that the proposed method allows access to waveform shape analysis for arteries of interest without location constraints. Furthermore, as is evident from the graphs, the error bars from five different measurements also provide valuable insights. With the MAXIM, the error range was ±0.46 from the average value. This indicates that there were cases where the 2nd harmonic peak was more significant than the 1st harmonic peak. In the case of BIOPAC, the error range was a maximum of ±0.16. In contrast, our device showed a deviation of a maximum of 0.1. Thus, our device, not only detects the various waveforms, but it also demonstrates an advantage in comparing the robustness of the harmonic waves and the ratio. Thus, the proposed device provides a solution that potentially allows convenient monitoring of arterial conditions at the point-of-care on all arteries without the need for users to set up separate signal processing systems.

All experiments were conducted in a laboratory at room temperature with the subject at rest and under the same conditions. Peak detection and ratio comparison were performed using the commercial software MATLAB R2021a. The method involved transforming each signal into the frequency domain and the frequency component with the highest peak, after the general respiratory frequency of 0.3 Hz, was analyzed. This frequency was defined as the first harmonic and, based on this first harmonic frequency, we defined the second harmonic frequency. Then, the amplitude of the second harmonic was investigated. Subsequently, a ratio comparison was conducted by comparing the amplitudes at these two frequencies. For the system comparison, NICO100C (BIOPAC Systems Inc., Goleta, CA, USA) and MAX30009EVKIT (Analog Devices, Inc.) were used. For the BIOPAC setup, an injection frequency of 12.5 kHz was implemented and a Lowpass filter (LPF) with a cutoff frequency of 10 Hz was added. For the setup from Maxim, an injection frequency of 9.984 kHz and a digital LPF with a cutoff frequency of 6.24 Hz were added after signal acquisition. However, in the circuit designed for this research, analog filters were added as noted above (bandpass filter of 0.3 Hz to 3 Hz and low-pass filter with a cutoff frequency of 3 Hz) but no additional digital filter was added. All detailed numerical values are available in Table 2.

### 3.3. Bioimpedance Circuit and Node-by-Node Signal Examples

**Figure 10:**
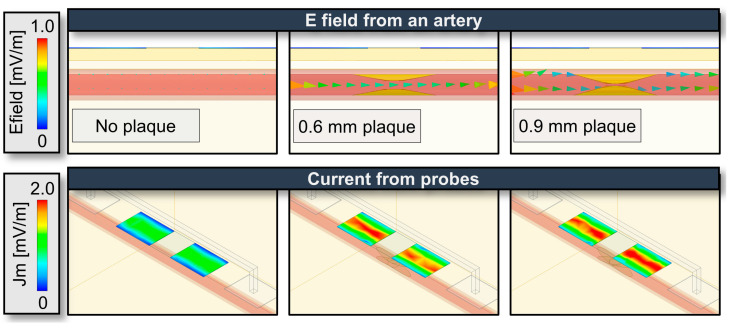
HFSS simulation depicts bioimpedance signal changes with varying plaque sizes.

**Figure 11:**
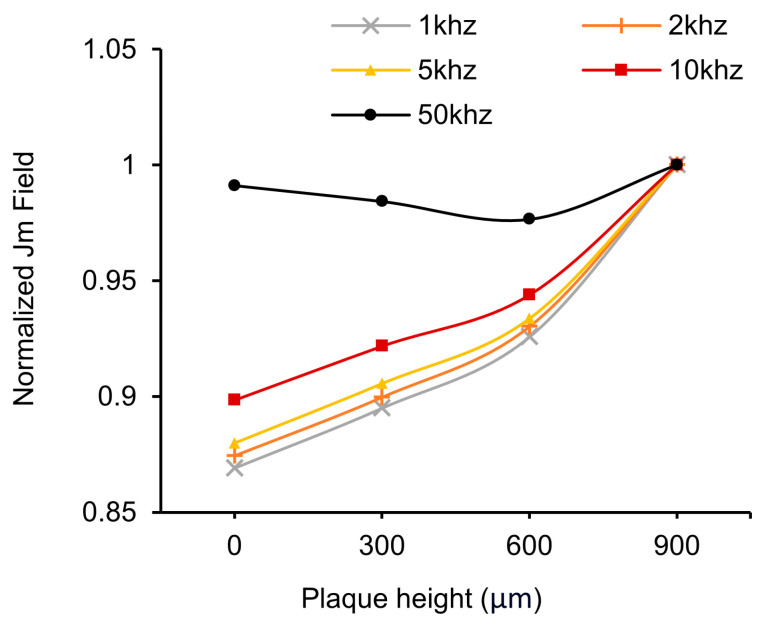
Normalized data comparison of average induced amount of current from the voltage detection electrodes with injection frequency.

**Figure 12:**
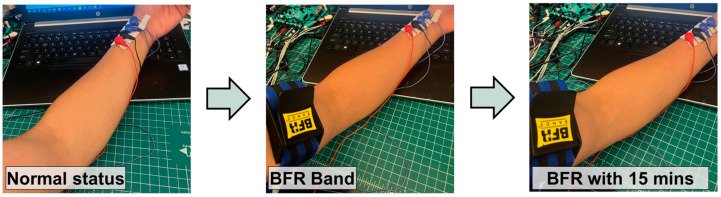
Utilization of the blood flow restriction (BFR) band to mimic PAD conditions.

**Figure 13:**
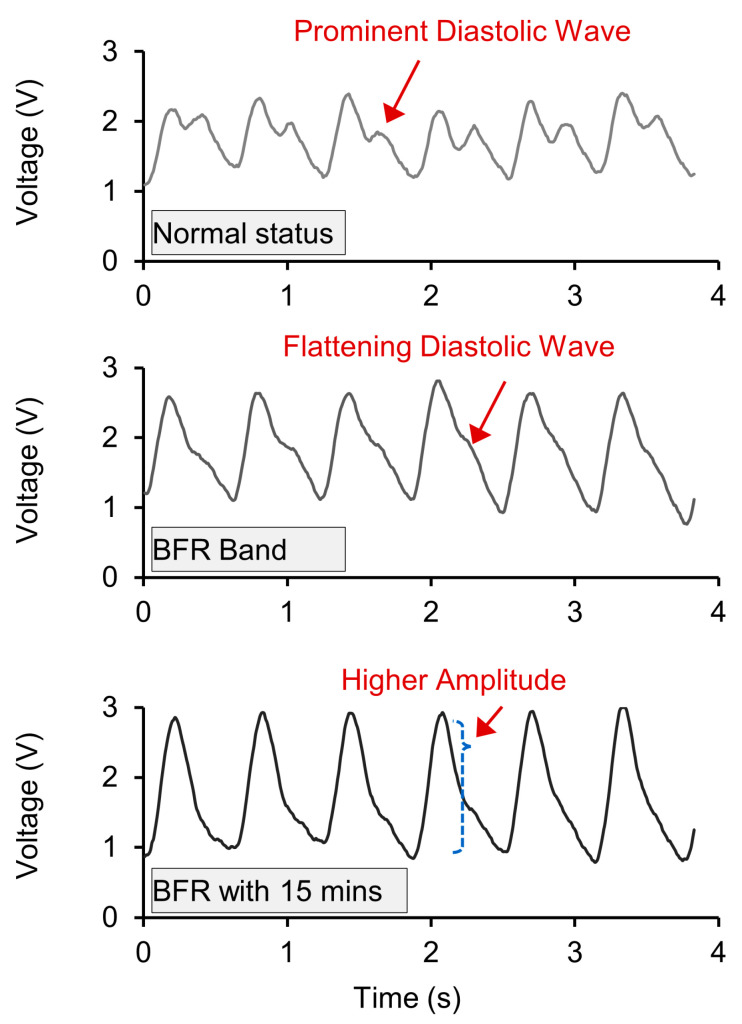
Morphological comparisons of arterial pulse signals from each stage of the experiment.

The method for monitoring PAD with morphological analysis using the first and second harmonic is further illustrated in Figure 10, Figure 11, Figure 12 and Figure 13. As shown in the HFSS simulation in Figure 10, when the height of the plaque in the blood vessel increases and disrupts the flow, in general, a stronger E field can be observed.

Figure 11 represents the relative changes of current from the voltage detection electrodes as the size of the plaque increases. It can be observed that at relatively low frequencies (1 kHz, 5 kHz, 10 kHz), the amount of induced current increases with the size of the plaque. However, as previously seen in Figure 6c, the 50 kHz modulation was shown to penetrate the tissue beyond the artery and hence has a relatively weak change with plaque height and flow, making it unsuitable for PAD’s morphological analysis. The formula and setup for this experiment are presented in Supplementary Figure S14.

When there is interference in blood flow due to PAD, not only does the bioimpedance signal increase but also there occurs changes in the shape of the pulse. According to studies using Doppler Velocimetry, the blood pressure signal during systole and diastole becomes flattened [39]. In particular, the diastolic signal decreases. To quantitatively analyze this, we utilize the fact that systolic blood pressure is represented more by the first harmonic frequency of the pulse, while diastolic blood pressure is represented more by the second harmonic frequency [56,76]. By examining the trend in the changes in the ratio between these two, we can potentially utilize this to monitor the progress of PAD. In this experimental study, in order to mimic the symptoms of PAD, a blood flow restriction (BFR) band was used on the upper arm and the radial artery bioimpedance was monitored for three conditions (normal, immediately after wearing the BFR band, and after 15 min). Inducing blood flow impedance using the BFR band has been shown to be a model for PAD [38]. The major difference between blood flow restriction (BFR) and actual PAD is venous blood return. In BFR, venous blood return is restricted, whereas it is not restricted in PAD [37,38].

In Figure 12 the experimental setup is shown and Table 3 shows the comparison of the first and second harmonic frequency components for each of the three experimental conditions. The experiments consisted of a total of six readings, with each reading including the recording of a 20-s signal. As evident from the experiments, when the BFR band is applied, there is an overall increase in the waveform with, on average, an increase in the first harmonic, an initial increase in the second harmonic amplitude after applying BFR and then a decrease after 15 min yielding an overall decrease in the harmonic ratio. Comparing the actual ratios, in a normal state, the ratio of the first to second harmonic amplitudes is 0.43 and, when blood flow is restricted through the BFR band, this ratio decreases over time from 0.3 to 0.233 with an error range of approximately ±0.06, which is relatively small compared to the deviation at each stage. This indicates that vascular conditions potentially can be predicted based on this wave analysis in terms of harmonic ratio, however, the standard errors are relatively large and the sample set is small.

In Figure 13, the raw data from the bioimpedance device are shown for the cases with the BFR band. We were able to observe the variation in the waveform morphological shapes that were predicted to be seen in PAD and which are similar to the pathologic study on PAD using Doppler velocimetry [39]. Specifically, for normal status, clear systolic and diastolic waves can be observed. However, as the time wearing the BFR band increased, the diastolic wave became less pronounced. After 15 min of applying the BFR band, it was difficult to visually identify the diastolic wave. A notable difference from the Doppler velocimetric test was that using bioimpedance for pulse measurement resulted in an increase in signal amplitude as the vascular stimulation intensified instead of flattening all the pulse waves [77].

## 4. Discussion

In this research, we developed a potential method for monitoring PAD at the point-of-care (POC) using a morphological approach to artery pulse signals. We used HFSS simulations to identify 10 kHz as the most sensitive injection frequency for vascular monitoring with our bioimpedance device. We designed, built, and tested a tetherless bioimpedance device that allowed for continuous monitoring of the ratio of the 1st and 2nd harmonics without spatial and temporal constraints. This capability enabled us to monitor trends in the harmonic ratio in vivo while mimicking PAD. Thus, overall, we anticipate that our device will contribute to facilitating PAD monitoring with a more accessible POC approach. Overall, our device exhibits the potential to enable both in-clinic and home monitoring for PAD due to its ability to monitor the harmonic ratio across arteries without modifying the settings, the ability to predict occlusion levels, its small form factor, and its ability for Bluetooth transmission to a phone or other device for final analysis. The device can then provide remote data transmission to healthcare professionals providing them access to up-to-date information, allowing timely intervention if concerning trends are detected. However, the system still needs much more human testing across thousands of patients and the development of the app. software to convert the harmonic ratios into actionable information for both the patient and health care provider. In future research, improving the signal accuracy could be explored through the use of Direct Digital Synthesis (DDS) or conducting studies to assess the signal accuracy as an AM circuit. Also, further discussion can be conducted on the utilization of more advanced circuits specifically for current pumping, such as expanding the output impedance [41]. Moreover, to enhance clinical feasibility, further research involving much larger numbers of diverse participants and a comparison between healthy subjects and PAD patients would be needed.

## Figures and Tables

**Table 1:** Comparative table for state-of-the-art techniques in similar domains.

Method	Objective Measurements	Cost Efficiency	Portable for POC	Ionizing Radiation
X-rays	Yes	No	No	Yes
MRI	Yes	No	No	No
CT	Yes	No	No	Yes
Blood test	Yes	No	No	No
Ankle-brachial index	No	Yes	Yes	No
Toe-branchial index	No	Yes	Yes	No
Toe pressure	No	Yes	Yes	No
Our Technology	Yes	Yes	Yes	No

**Table 2:** Quantitative details for device comparison.

Method	BIOPAC	MAX30009 EVKIT	Suggested Method
Injection Frequency	12.5 kHz	9.984 kHz	10 kHz
Injection Current amount	400 μA	96 μA	84.7 μA
Digital Filter Type	Low Pass Filter	Low Pass Filter	N/A
Cutoff Frequency	10 Hz	6.24 Hz	N/A
Measurement Time	30 s	30 s	30 s

**Table 3:** Assess harmonic wave composition under different experimental flows.

	1st Harmonic	2nd Harmonic	Harmonic Ratio (2nd/1st)
Normal	24.73 dB (±1.36 dB)	17.43 dB (±2.53 dB)	0.43 (±0.06)
BFR	30.03 dB (±0.83 dB)	19.43 dB (±2.53 dB)	0.30 (±0.05)
BFR 15 mins	30.03 dB (±0.83 dB)	17.17 dB (±3.36 dB)	0.23 (±0.06)

## Data Availability

The data presented in this study are available on request from the corresponding author.

## Supplementary Materials

The following supporting information can be downloaded at: https://www.mdpi.com/article/10.3390/bios14060286/s1, Figure S1: Simulation of unstable oscillation when using a Wien Bridge oscillator; Figure S2: Comparison circuit simulation of the original current pump and the improved current pump; Figures S3–S9: Altium schematic of the circuit used in the experiment; Figure S10: BOM (Bill of Materials) for the circuit used in the experiment; Figure S11: Model parameter values for different frequencies used in HFSS; Figure S12: Results of rotating the electrodes at various angles; Figure S13: Calculator method used in HFSS; Video S1: Demonstration video of the actual operation.
