# Supplementary material for: Development of a Tetherless Bioimpedance Device That Uses Morphologic Changes to Predict Blood Flow Restrictions Mimicking Peripheral Artery Disease Progression
Source: Biosensors (Basel). 2024 Jun 1;14(6):286. doi: 10.3390/bios14060286 (PMC11202059; doi:10.3390/bios14060286)

Supplementary Figure S1.

a Circuit diagram of the filter (Frequency 50kHz)

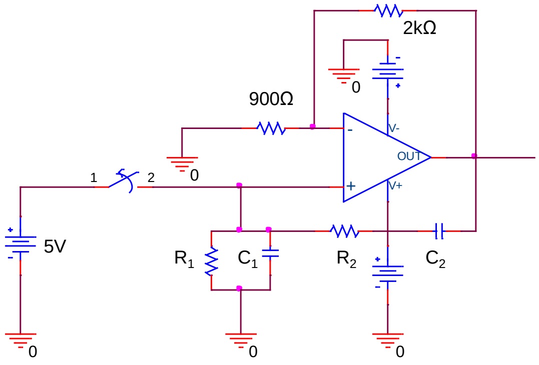

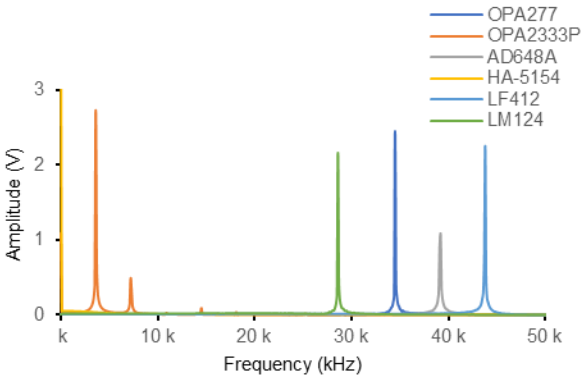

b Circuit diagram of the filter (Ideal Frequency 50kHz)

	**-10%**	**-5%**	**Exact value**	**+5%**	**+10%**
C_1	14.4nF	15.2nF	16nF	16.8nF	17.6nF
C_2	14.4nF	15.2nF	16nF	16.8nF	17.6nF
R_1	180Ω	190Ω	200Ω	210Ω	220Ω
R_2	1800Ω	190Ω	200Ω	210Ω	220Ω
Amplitude	N/A	2.05V	2.08V	2.53V	2.47V
Resonant Frequency	N/A	37kHz	35kHz	32kHz	30kHz

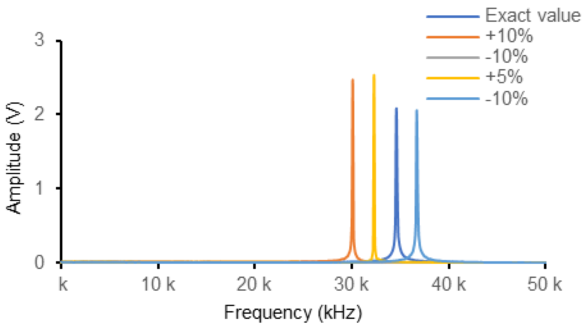

Supplementary Figure S2.

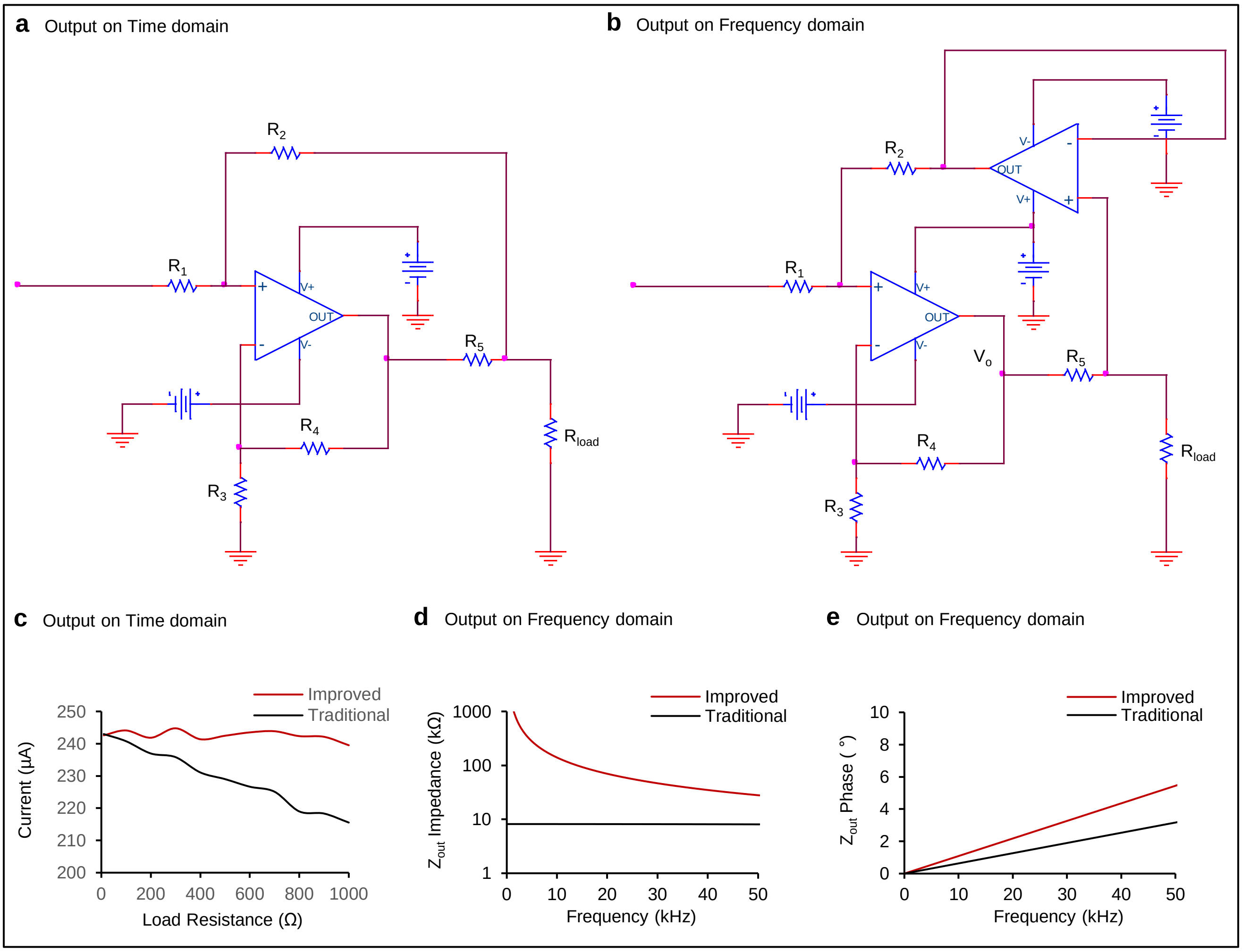

Supplementary Figure S3.

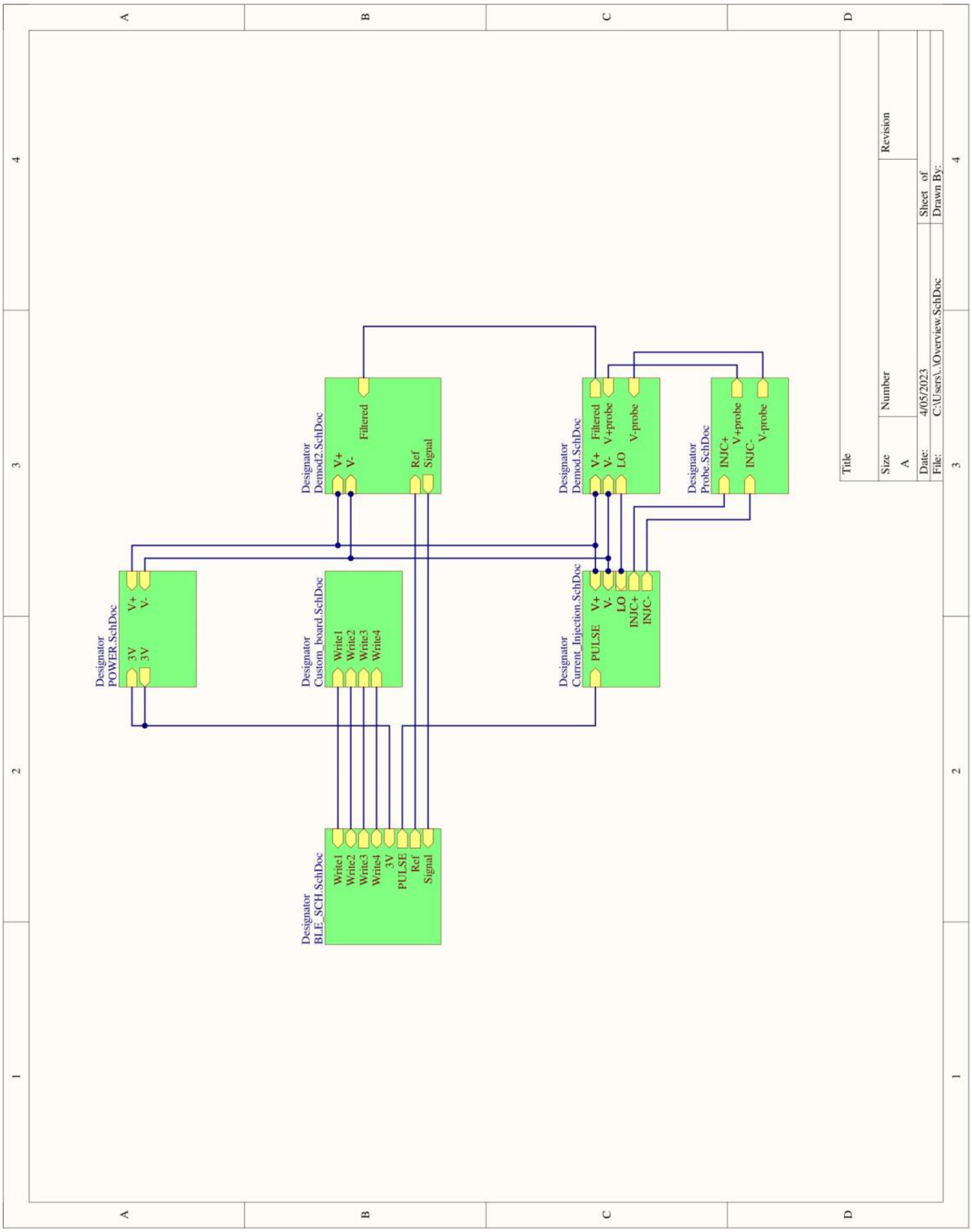

Supplementary Figure S4.

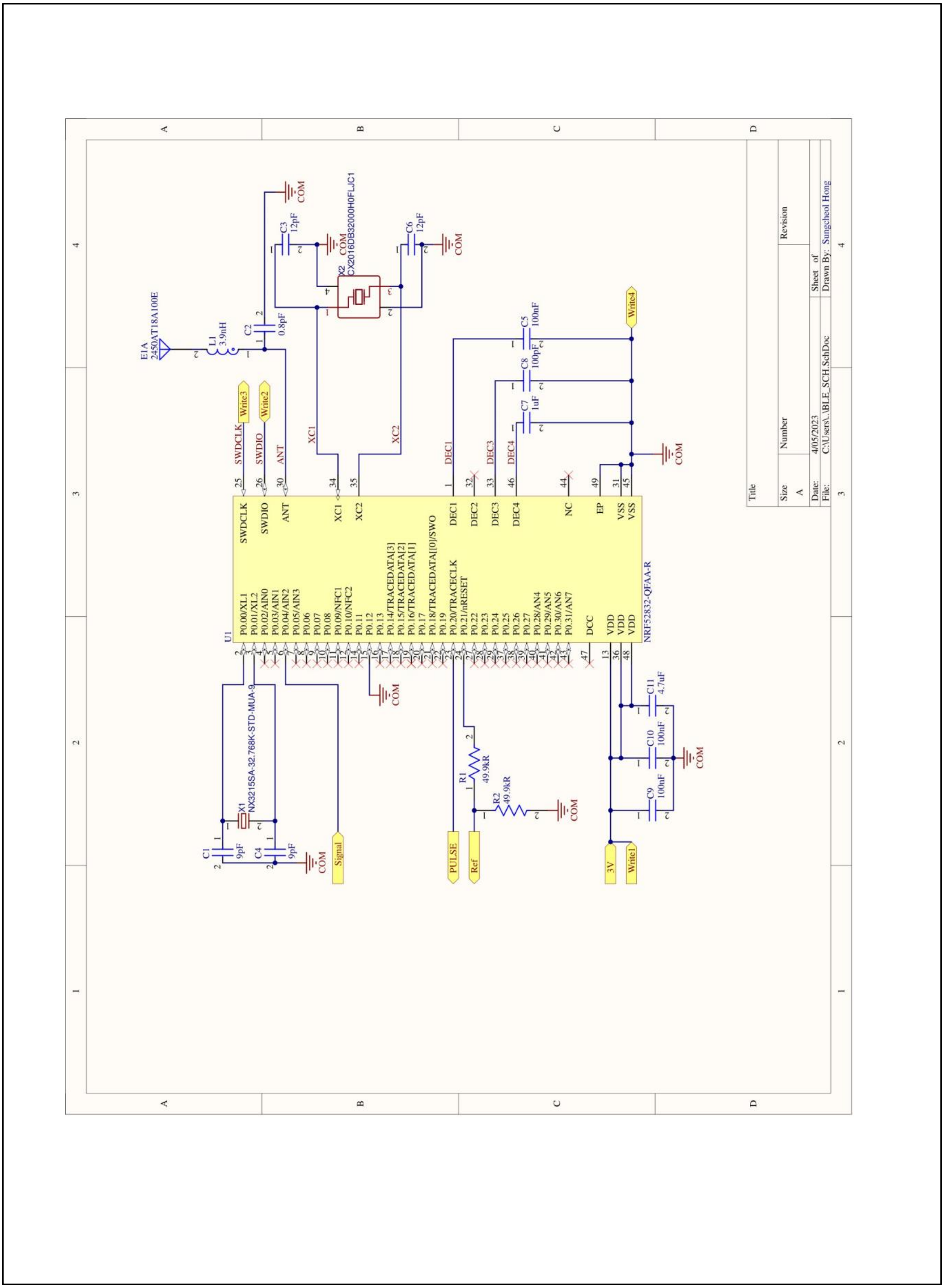

Supplementary Figure S5.

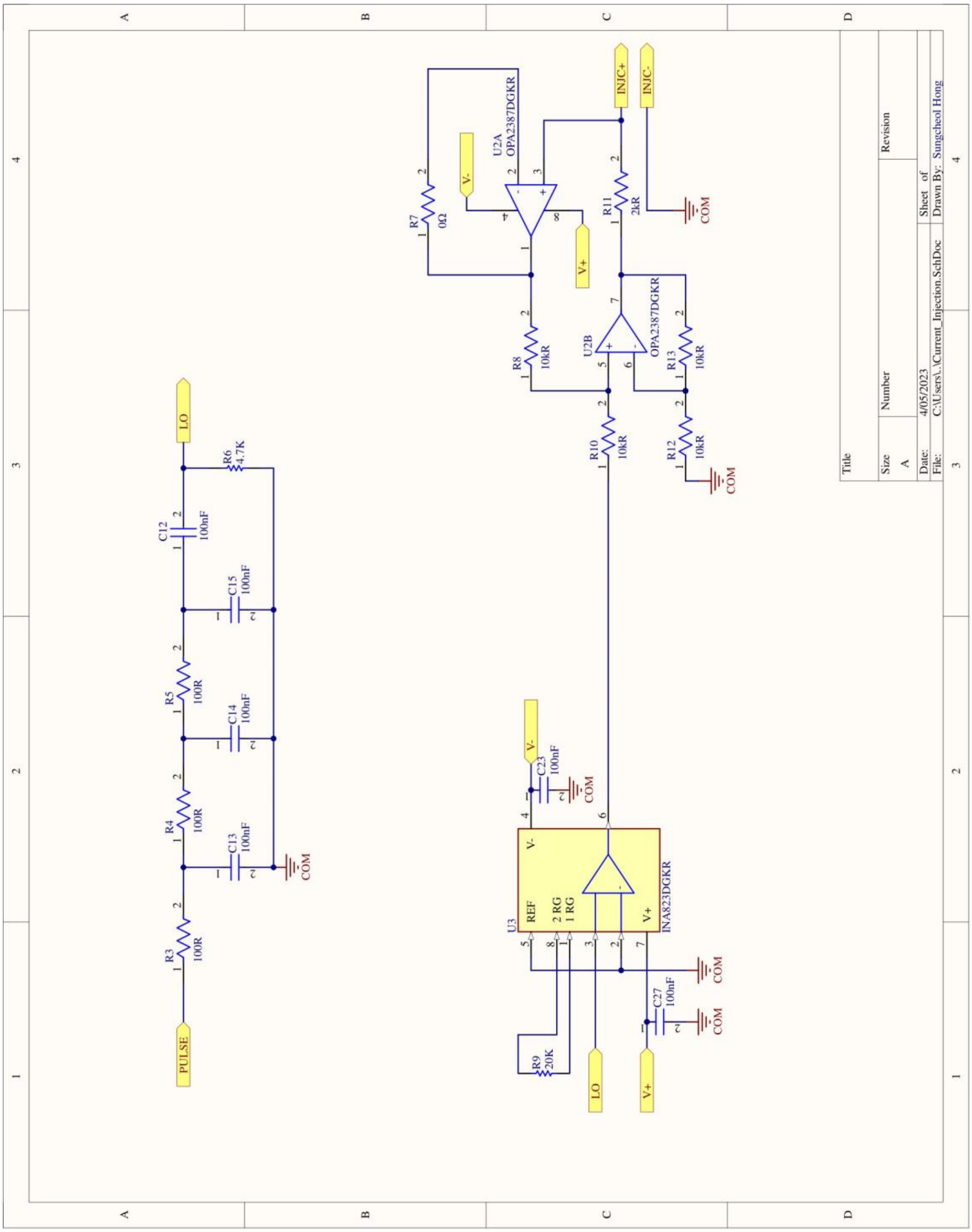

Supplementary Figure S6.

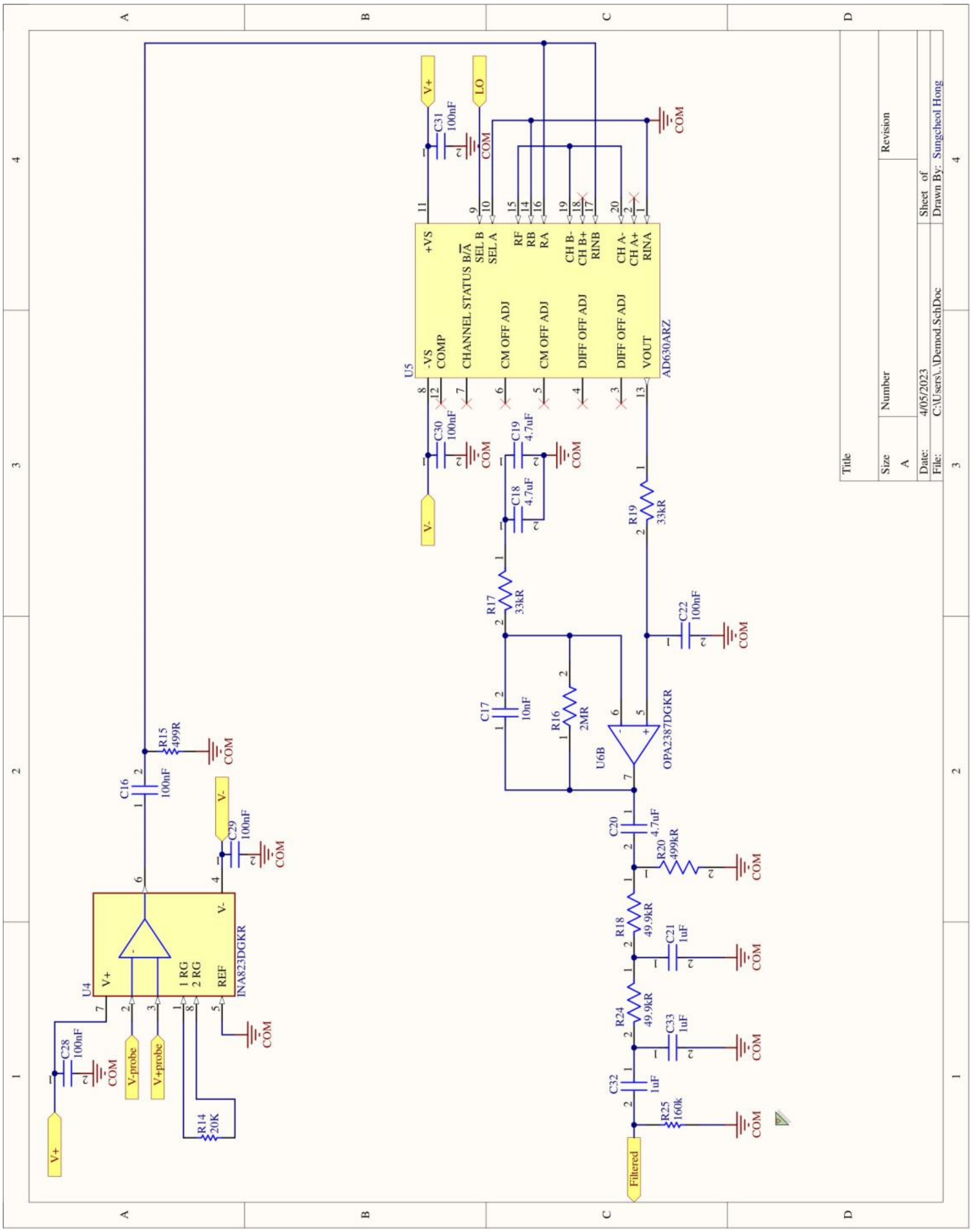

Supplementary Figure S7.

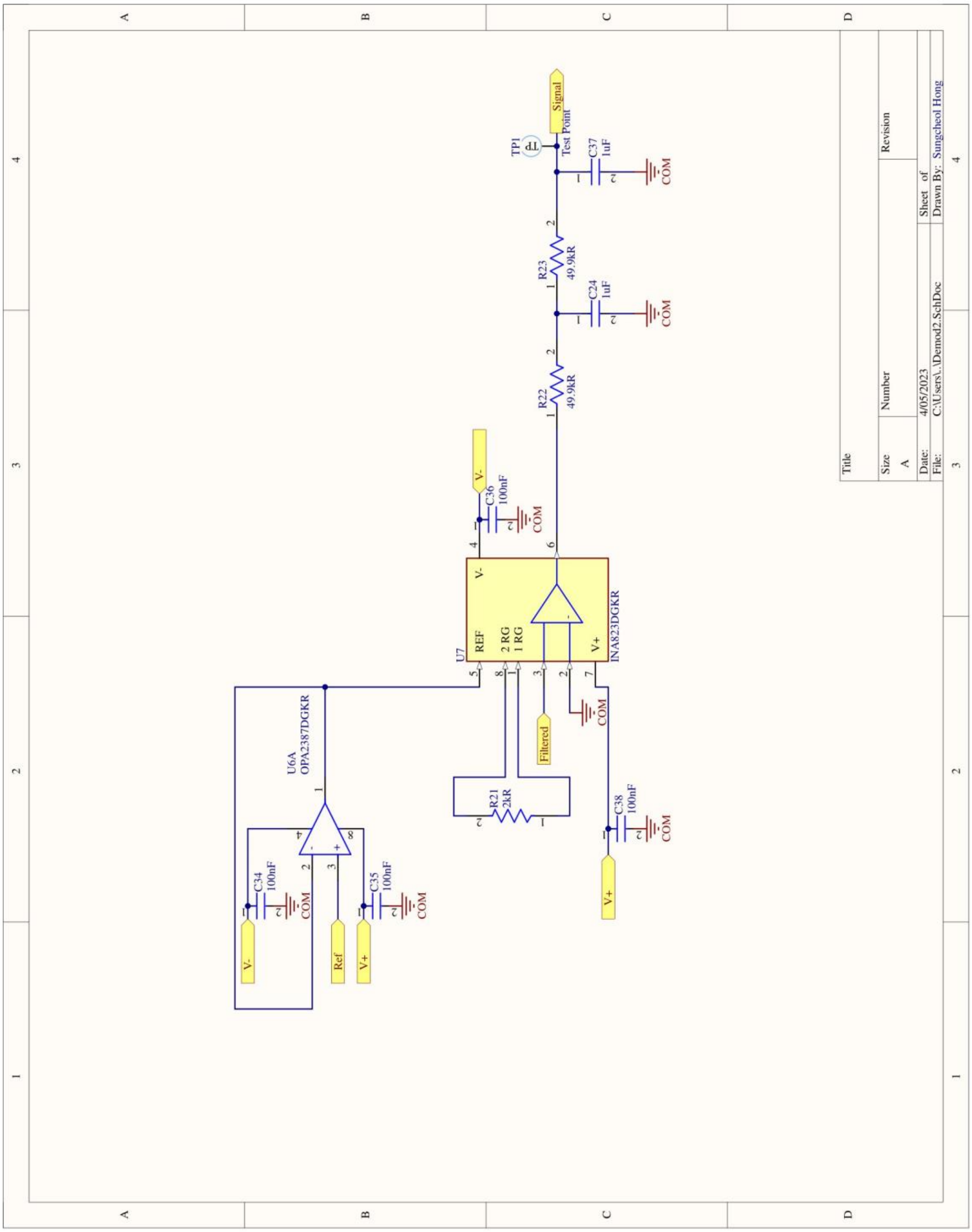

Supplementary Figure S8.

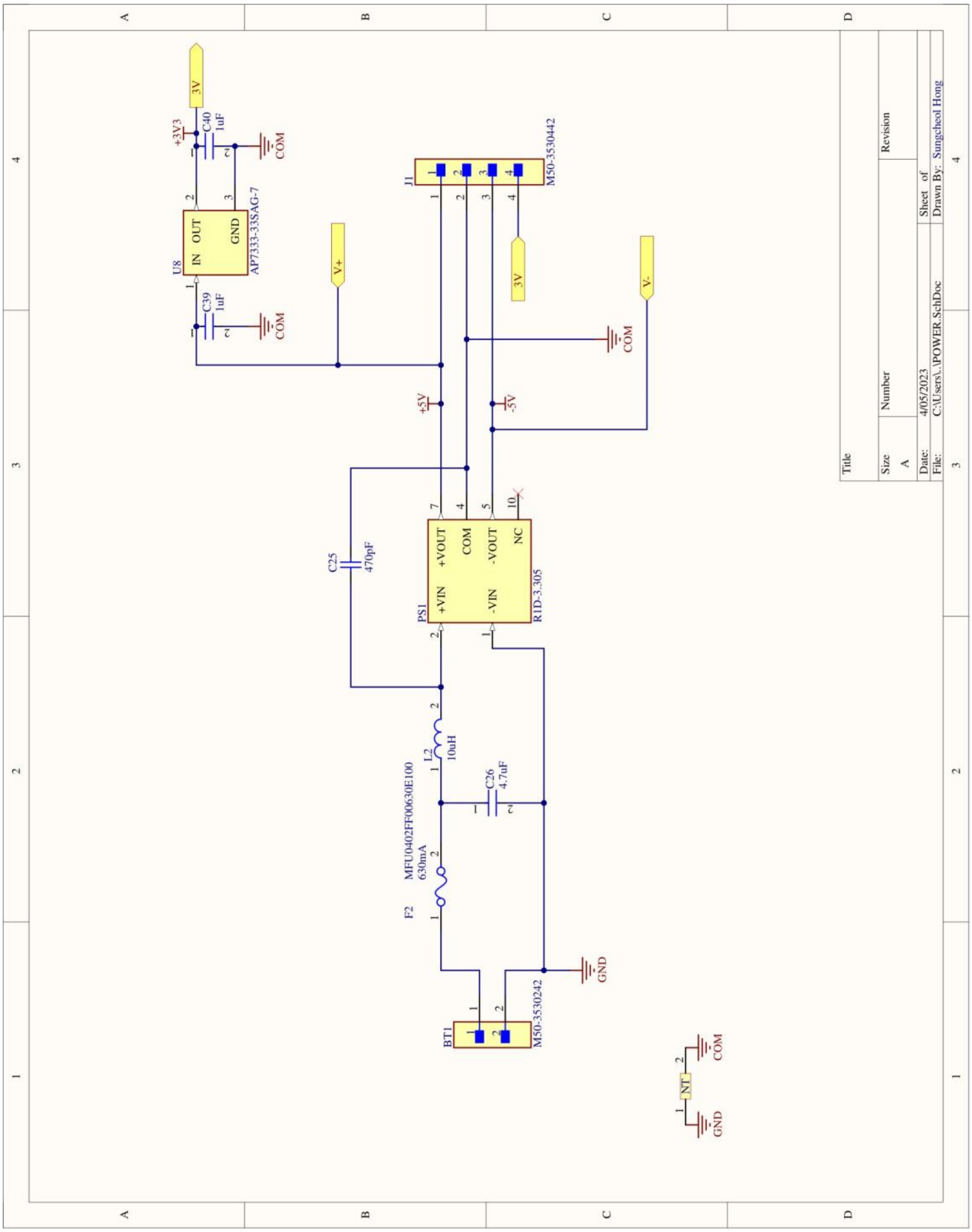

Supplementary Figure S9.

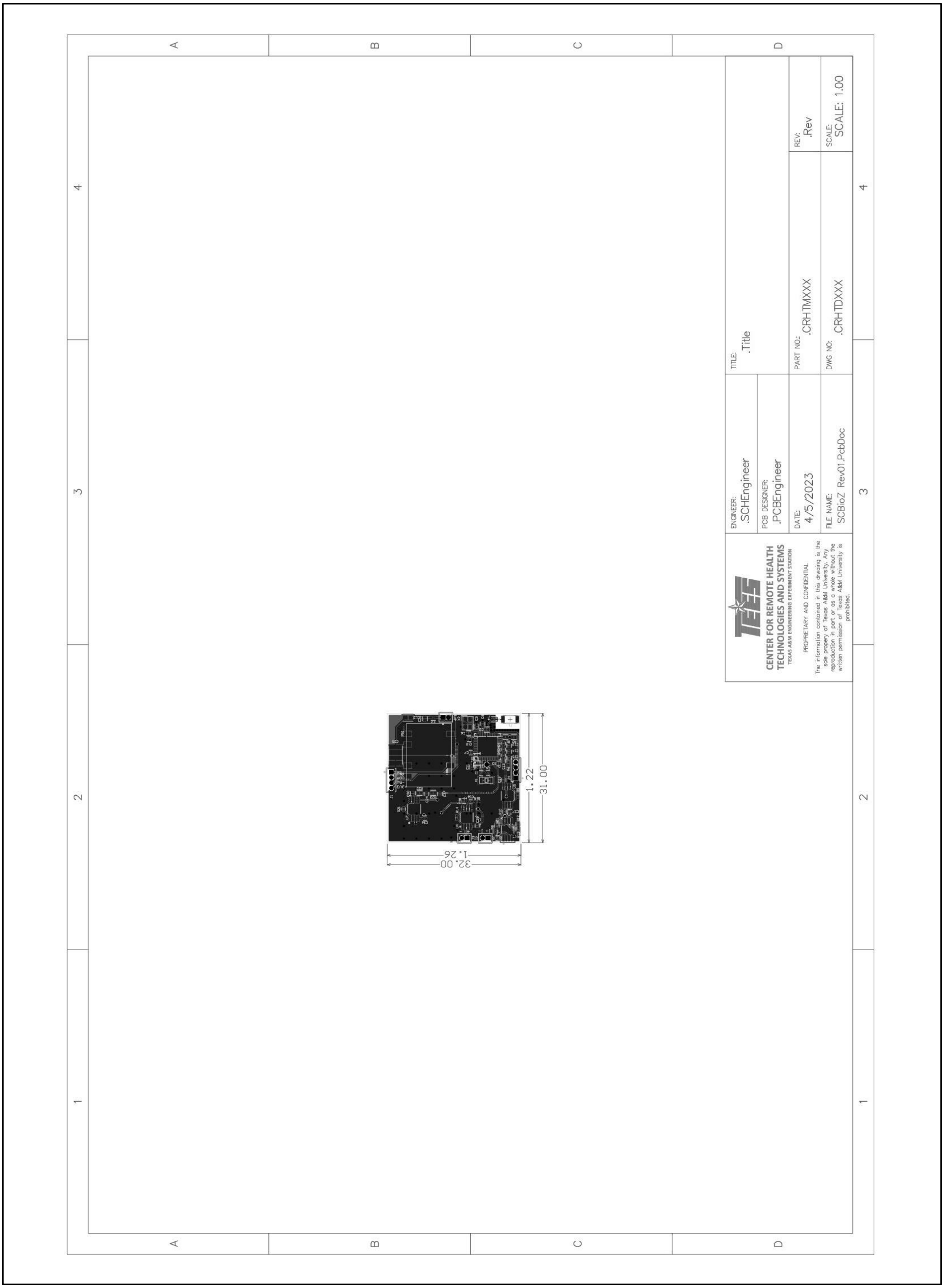

Supplementary Figure S10.

Line #	Name	Designator	Description	Footprint	Value	Quantity	Manufacturer 1	Manufacturer Part Number 1	Manufacturer Lifecycle 1	Supplier 1	Supplier Part Number 1	Supplier Unit Price 1	Supplier Subtotal 1
	Test Point	TP1		Thru_TP		1							
	RC0603J202CS	R11, R21	Res Thick Film 0201 2kΩ 5% 0.05W ±250ppm/°C Molded SMD T/R	FP-RC0603-MFG		2	Samsung	RC0603J202CS	Volume Production				
	RC0603F4993CS	R20	Res Thick Film 0201 499kΩ 1% 0.05W ±250ppm/°C Molded SMD T/R	FP-RC0603-IPC_B		1	Samsung	RC0603F4993CS	End of Life	Arrow Electronics	RC0603F4993CS	0.0532	0.0532
	RC0603F4992CS	R1, R2, R18, R22, R23, R24	Res Thick Film 0201 49.9kΩ 1% 0.05W ±250ppm/°C Molded SMD T/R	FP-RC0603-IPC_A		6	Samsung	RC0603F4992CS	Volume Production				
	RC0603F333CS	R17, R19	Res Thick Film 0201 33kΩ 1% 0.05W ±250ppm/°C Molded SMD T/R	FP-RC0603-MFG		2	Samsung	RC0603F333CS	Volume Production				
	RC0603F103CS	R8, R10, R12, R13	Res Thick Film 0201 10kΩ 1% 0.05W ±250ppm/°C Molded SMD T/R	FP-RC0603-IPC_C		4	Samsung	RC0603F103CS	Not Recommended for New Design	Arrow Electronics	RC0603F103CS	0.0444	0.1776
	RC0603F101CS	R3, R4, R5	Res Thick Film 0201 100Ω 1% 0.05W ±250ppm/°C Molded SMD T/R	FP-RC0603-MFG		3	Samsung	RC0603F101CS	Volume Production	Arrow Electronics	RC0603F101CS	0.0906	0.2718
	RC0201JR-070RL	R7	RES SMD 0 OHM JUMPER 1/20W 0201	FP-RC_L0201-MFG	0Ω	1	Yageo	RC0201JR-070RL	Volume Production	Digi-Key	311-0.0NCT-ND	0.1	0.1
	R1D-3.305	PS1	1W DC/DC-Converter "ECONOLINE" SMD 1kV unreg; V. In: 3.3; V. Out: +/-05; mA. Out: +/-100	CONV_R1D-3.305		1				Mouser	919-R1D-3.305		
	OPA2387DGKR	U2, U6	ULTRA-HIGH PRECISION, ZERO-DRIFT	FP-DGK0008A-IPC_B		2	Texas Instruments	OPA2387DGKR		Mouser	595-OPA2387DGKR	4.13	8.26
	NX3215SA-32.768K-STD-MUA-9	X1		NX3215SA		1							
	NRF52832-QFAA-R	U1	Multiprotocol Bluetooth Smart, ANT/ANT+ and Proprietary System-on-Chip, 2.4 GHz, 256 KB FLASH, 1.7 to 3.6 V, -40 to 85 degC, 48-Pin QFN, RoHS, Tape and Reel	NRSC-QF-48_L		1	Nordic Semiconductor	NRF52832-QFAA-R	Volume Production	Mouser	949-NRF52832-QFAA-R	5.31	5.31
	MFU0402FF00630E100	F2	Surface Mount Fuses .63A 32V VERY FAST ACTING	FP-ERBRD-MFG	53mΩ	1	Panasonic	ERB-RD2R00X	Volume Production	Arrow Electronics	ERB-RD2R00X	0.2293	0.2293
	MCR006YRTJ205	R16	Thick Film Chip Resistor, 0201, 2MΩ, 5%, 200ppm/°C, 0.05W, 25V	FP-MCR006-IPC_C		1	Rohm	MCR006YRTJ205	Volume Production	Digi-Key	RHM2MCCDKR-ND	0.1	0.1
	M50-3530442	J1, J4	CONN HEADER VERT 4POS 1.27MM	FP-M50-3530442-MFG		2							
	M50-3530242	BT1, J2, J3	CONN HEADER VERT 2POS 1.27MM	FP-M50-3530242-MFG		3							
	LQP03TN3N9B02D	L1	Film type RF Inductor 3.9nH ±0.1nH 400mA 0.3Ω 0201 (0603)	FP-LQP03TG_02-IPC_A		1	Murata	LQP03TN3N9B02D	Volume Production	Arrow Electronics	LQP03TN3N9B02D	0.0925	0.0925
	LQM18DN100M70L	L2	Multilayer type Inductor for Power Lines 10uH ±20% @1MHz, 300mA, Max DCR 1.365Ω, SRF 32MHz	FP-LQM18DN_70-IPC_B		1	Murata	LQM18DN100M70L		Newark	14AJ2370	0.12	0.12
	INA823DGKR	U3, U4, U7	IC INST AMP 1 CIRCUIT 8VSSOP	FP-DGK0008A-MFG		3	Texas Instruments	INA333AIDGKT	Volume Production				
	GRM0335C1H101JA01D	C8	Chip Multilayer Ceramic Capacitors for General Purpose, 0201, 100pF, C0G, 30ppm/°C, 5%, 50V	FP-GRM033-0_03-IPC_C		1	Murata	GRM0335C1H101JA01D		Farnell	2494202	0.0057	0.05702
	GRM035R60J475ME15D	C11, C18, C19, C20, C26	Chip Multilayer Ceramic Capacitors for General Purpose, 0201, 4.7uF, X5R, 15%, 20%, 6.3V	FP-GRM035-0_09-IPC_B		5	Murata	GRM035R60J475ME15D	Volume Production	Mouser	81-GRM035R60J475ME5D	0.112	1.12
	GRM033R61A104KE15D	C5, C9, C10, C12, C13, C14, C15, C16, C22, C23, C27, C28, C29, C30, C31, C34, C35, C36, C38	Chip Multilayer Ceramic Capacitors for General Purpose, 0201, 0.10uF, X5R, 15%, 10%, 10V	FP-GRM033-0_03-IPC_C		19	Murata	GRM033R61A104KE15D	Volume Production	Newark	45Y5193	0.002	0.038
	CX2016DB32000H0FLJC1	X2		XTAL32M		1							
	CRCW0201499RFKED	R15		RESC0603X28X15ML05T05		1	Vishay Dale	CRCW0201499RFKED	Volume Production	Newark	65AC3339	0.022	0.022
	CRCW020120K0FKED	R9, R14		RESC0603X28X15ML05T05		2	Vishay Dale	CRCW020120K0FKED	Volume Production	Arrow Electronics	CRCW020120K0FKED	0.2683	0.5366
	CRCW02014K70FNED	R6		RESC0603X28X15NL05T05		1	Vishay	CRCW02014K70FNED	Volume Production	Arrow Electronics	CRCW02014K70FNED	0.0949	0.0949
	CL03C090CA3GNNC	C1, C4	Cap Ceramic 9pF 25V C0G ±0.25pF Pad SMD 0201 +125°C T/R	FP-CL03-IPC_C		2	Samsung	CL03C090CA3GNNC	Volume Production	Avnet	CL03C090CA3GNNC	0.00355	35.5
	CL03C0R8BA3GNNC	C2	Cap Ceramic 0.8pF 25V C0G ±0.1pF Pad SMD 0201 +125°C T/R	FP-CL03-IPC_A		1	Samsung	CL03C0R8BA3GNNC	Volume Production	Digi-Key	1276-1380-2-ND	0.01455	145.5
	CL03A105MP3ZSNH	C7, C21, C24, C32, C33, C37, C39, C40	Cap Ceramic 1uF 10V X5R ±20% Pad SMD 0201 +85°C T/R	FP-CL03-IPC_C		8	Samsung	CL03A105MP3ZSNH	New Product	Arrow Electronics	CL03A105MP3ZSNH	0.161	1.61
	Cap	C25	Capacitor, Film, SFTY 1812 470pF X1 250 / Y2 250 C0G, Reel	C_1812	470pF	1				Mouser	80-CAS18C471JAGFC		
	C0603C0G1H120J030BA	C3, C6	Multilayer Ceramic Capacitors 12pF ±5% 50V C0G SMD 0201	FP-C0603-030-0_03-IPC_B		2	TDK	C0603C0G1H120J030BA	Not Recommended for New Design	Mouser	810-C0603C0G1H120J	0.1	0.2
	AP7333-33SAG-7	U8	IC REG LINEAR 3.3V 300MA SOT23-3	FP-SOT23-MFG		1	Diodes	AP7333-33SAG-7	Volume Production				
	AD630ARZ	U5	Balanced Modulator/Demodulator, 2MHz Max RF Frequency, RW-20, 19 Tube	ADI-RW-20_L		1	Analog Devices	AD630ARZ	Volume Production	Digi-Key	505-AD630ARZ-ND	36.05	36.05
	2450AT18A100E	E1	Ceramic Antenna, 2.45 GHZ, 2 W, -40 to 125 degC, 2-Pin SMD, RoHS, Tape and Reel	JT-2450AT18A100-2_V		1	Johanson	2450AT18A100E	Volume Production	Digi-Key	712-1005-1-ND	0.59	0.59
	0201ZC103KAT2A	C17	General Purpose Ceramic Capacitor, 0201, 10nF, 10%, X7R, 15%, 10V	FP-0201-L_0_6_0_09-W_0_3-IPC_C		1	Kyocera AVX	0201ZC103KAT2A	Volume Production	Newark	15P4031	0.077	0.077
	160k	R25		RESC0603X28X15NL05T05		1	Vishay Dale	CRCW0201160KJNED	Volume Production				

Supplementary Figure S11.

	Permittivity	Conductivity [S/m]
Artery Wall	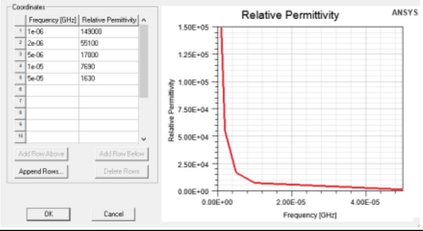	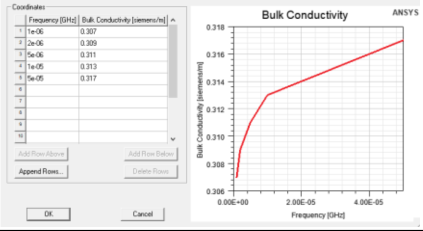
Blood	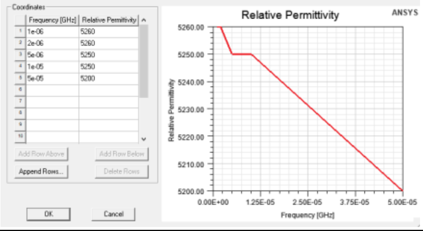	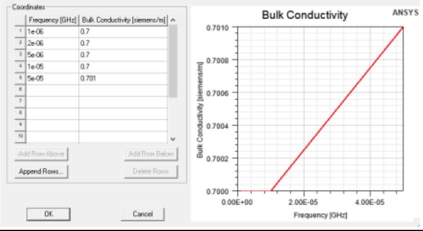
Fat (Tissue)	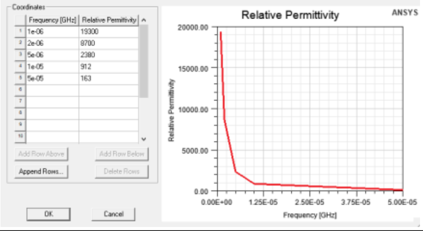	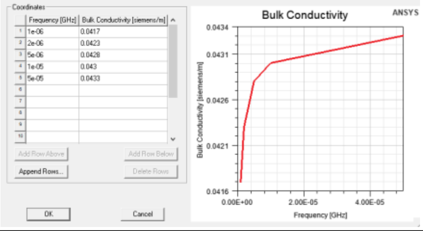
Skin	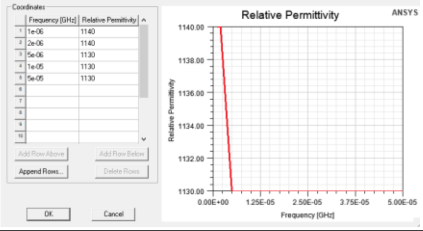	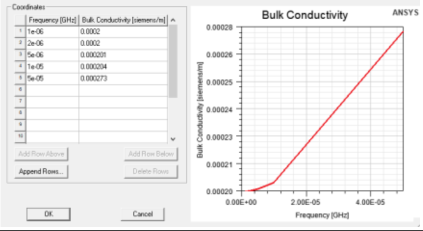

	Frequency	Permittivity	Conductivity [S/m]
Artery wall	1kHz	149000	0.307
	5kHz	17000	0.311
	10kHz	7690	0.313
	50kHz	1630	0.317
Blood	1kHz	5260	0.7
	5kHz	5250	0.7
	10kHz	5250	0.7
	50kHz	5200	0.701
Fat (Tissure)	1kHz	19300	0.0417
	5kHz	2380	0.0428
	10kHz	912	0.043
	50kHz	163	0.0433
Skin	1kHz	1140	0.0002
	5kHz	1130	0.000201
	10kHz	1130	0.000204
	50kHz	1130	0.000273

Supplementary Figure S12.

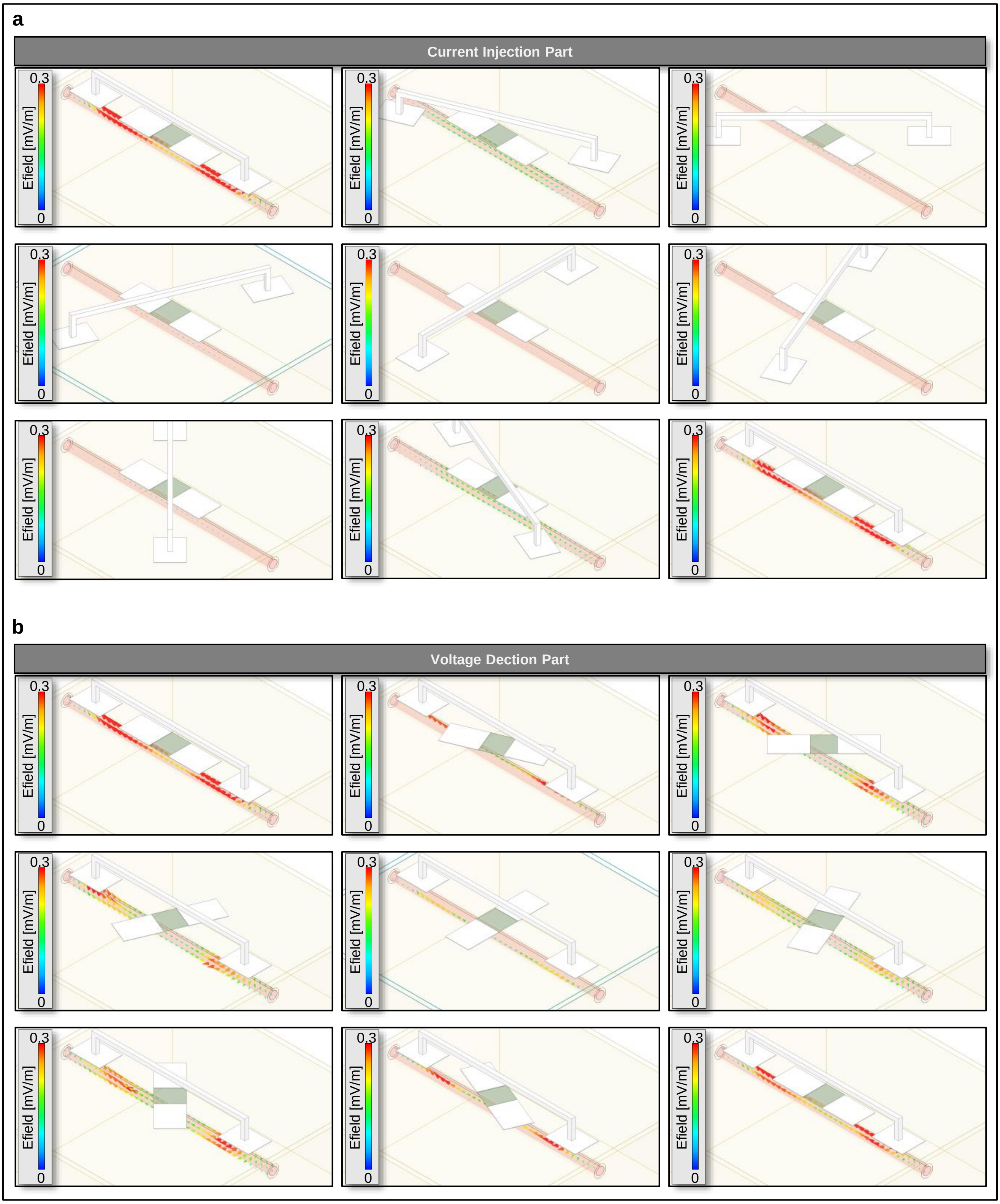

Supplementary Figure S13.

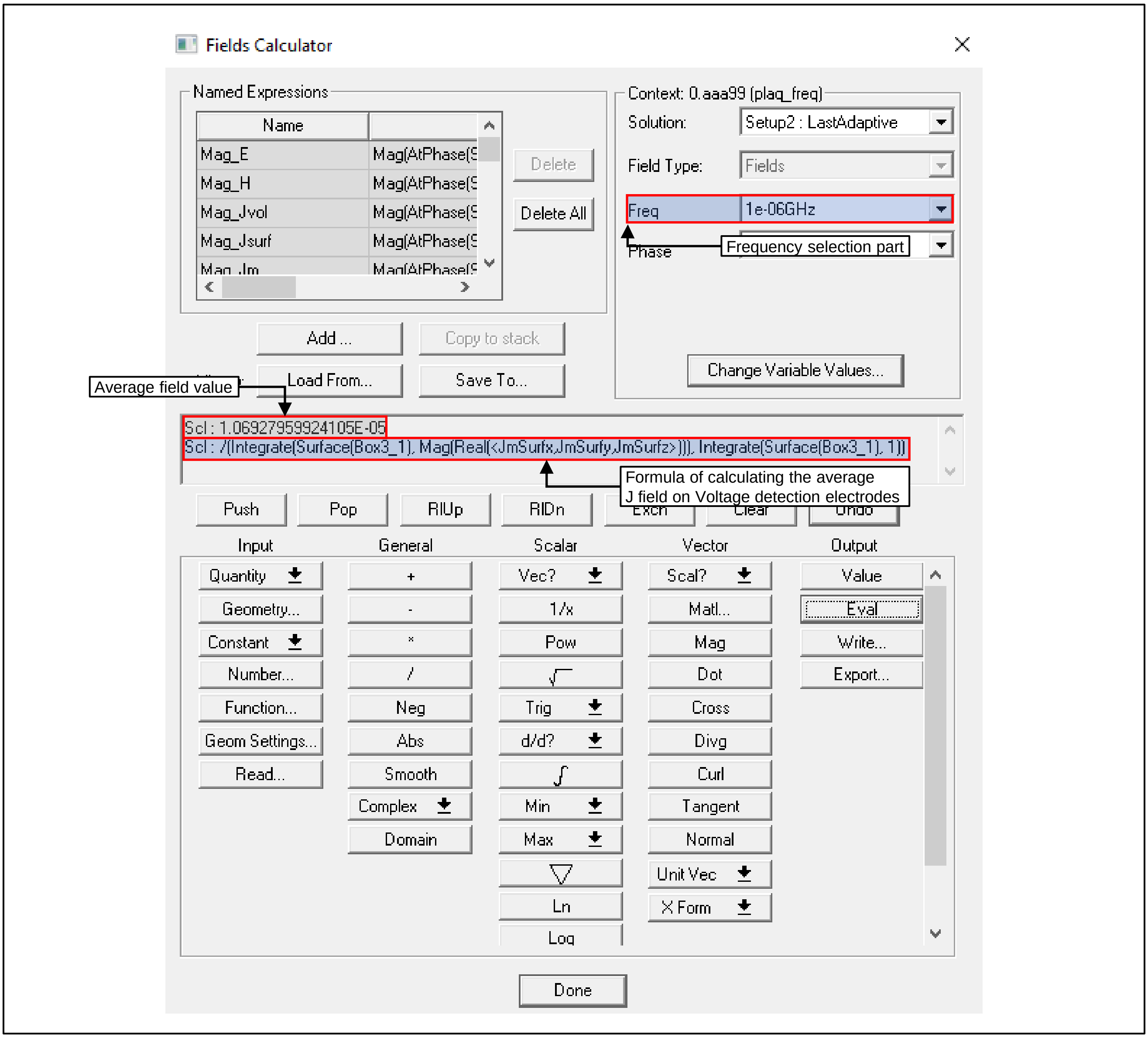

Supplement: Supplementary file 1 [file biosensors-14-00286-s001.zip › Supplementary_Information.pdf]
